# Supplementary material for: Betrixaban activates cGAS and ERVs to promote dual nucleic-sensing antiviral immunity
Source: EMBO Mol Med. 2026 Mar 23;18(5):1563–91. doi: 10.1038/s44321-025-00356-7 (PMC13179341; doi:10.1038/s44321-025-00356-7)
Supplement: Supplementary file 3 — Appendix [file 44321_2025_356_MOESM3_ESM.pdf]

Appendix for "Betrixaban Activates cGAS and ERVs to Promote Dual Nucleic-Sensing Antiviral Immunity"

Table of Contents

1. **Appendix Figure S1** 2

2. **Appendix Figure S2** 3

3. **Appendix Table S1** 4

Appendix Figure S1

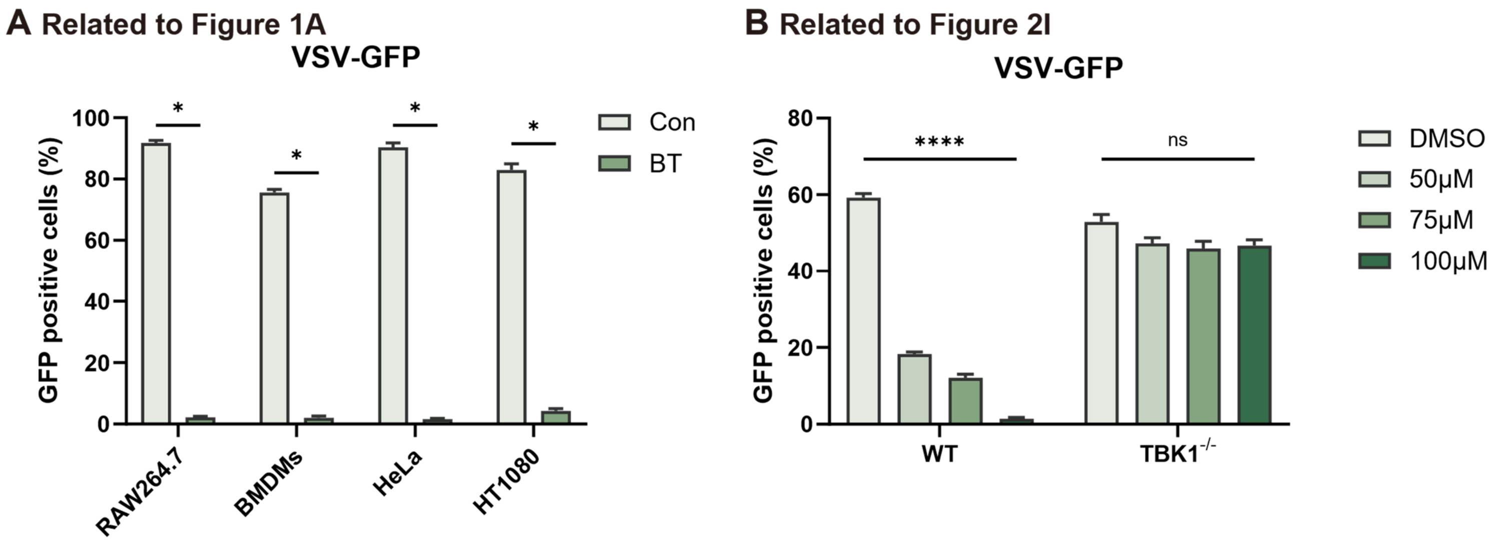

Appendix Figure S1. Quantification of the presented Flow cytometry.

(A-B) Flow cytometry quantification of Figure 1A (A) and Figure 2I (B). Data are shown as mean ± SEM. N.S., not significant, $p > 0.05$; *$p < 0.05$; **$p < 0.01$; ****$p < 0.0001$.

Appendix Figure S2

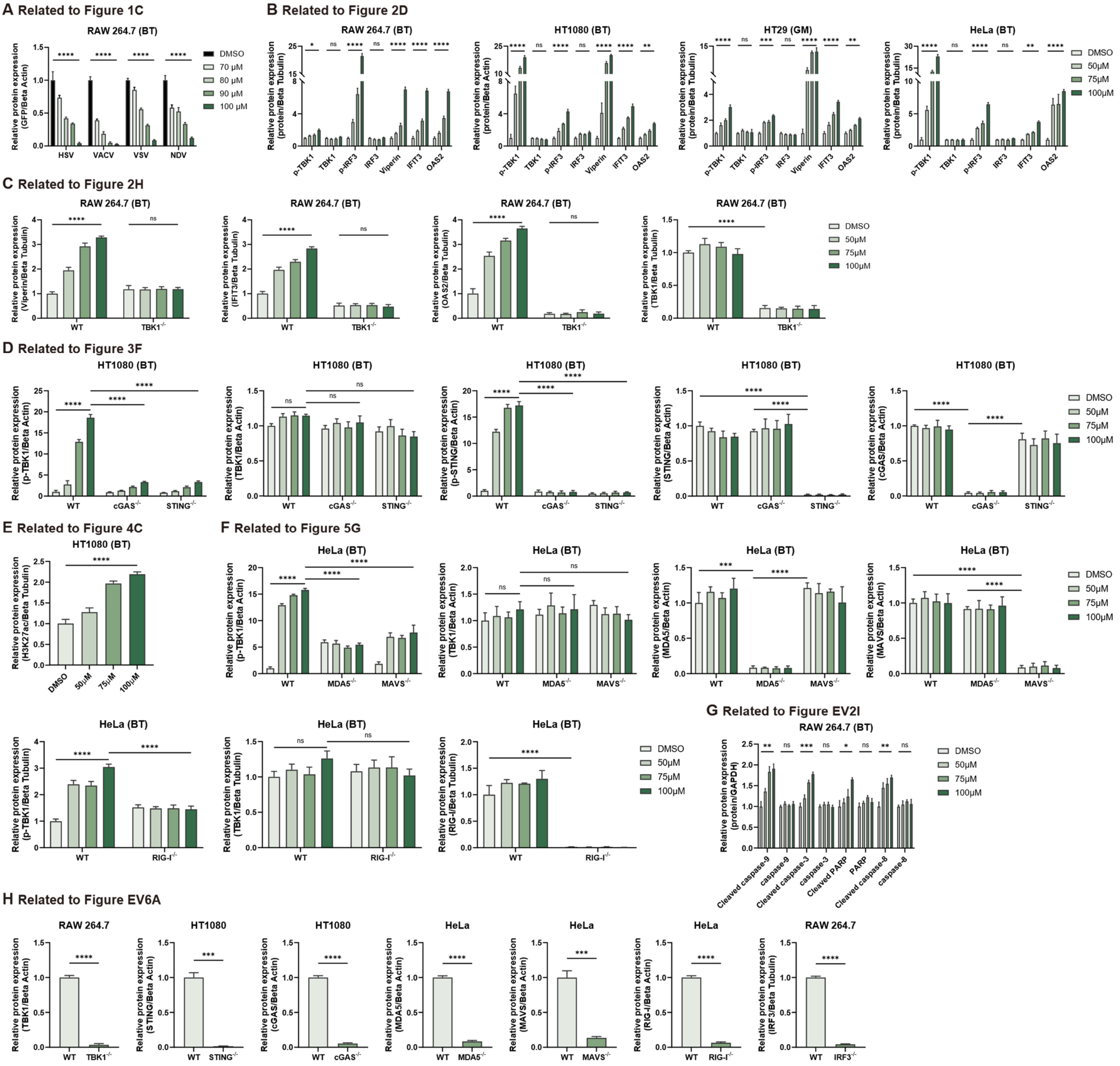

Appendix Figure S2. Quantification of the presented Western blots.

(A-H) quantification of band intensities in Figure 1C (A), Figure 2D (B), Figure 2H (C), Figure 3F (D), Figure 4C (E), Figure 5G (F), Figure EV2I (G) and Figure EV6A (H). Densitometry was performed in ImageJ 1.51 using fixed rectangular ROIs with local background subtraction. Target proteins were normalized to loading control and then to the vehicle group, which was set to 1.0. Data are shown as mean ± SEM (n = 3 independent experiments). N.S., not significant, $p > 0.05$; *$p < 0.05$; **$p < 0.01$; ***$p < 0.001$; ****$p < 0.0001$.

Appendix Table S1. Exact *P* value.

Figure	Statistical method	Groups	Exact *P* value
Figure 1D	Unpaired t test	EMCV in Con vs EMCV in BT	0.0065
Figure 1D	Unpaired t test	HSV in Con vs HSV in BT	0.0001
Figure 1D	Unpaired t test	IAV in Con vs IAV in BT	0.041
Figure 1D	Unpaired t test	MHV in Con vs MHV in BT	0.0039
Figure 1D	Unpaired t test	VSV in Con vs VSV in BT	0.0093
Figure 1D	Unpaired t test	BA.2.86 in Con vs BA.2.86 in BT	<0.0001
Figure 1D	Unpaired t test with Welch's correction	D614G in Con vs D614G in BT	0.0034
Figure 1D	Unpaired t test	BA.2 in Con vs BA.2 in BT	<0.0001
Figure 1D	Unpaired t test with Welch's correction	BA.1 in Con vs BA.1 in BT	0.0049
Figure 1D	Unpaired t test	Delta in Con vs Delta in BT	<0.0001
Figure 1E	Unpaired t test	EMCV in Con vs EMCV in BT	0.0068
Figure 1E	Unpaired t test	HSV in Con vs HSV in BT	0.0103
Figure 1E	Unpaired t test	IAV in Con vs IAV in BT	0.0048
Figure 1E	Unpaired t test	VSV in Con vs VSV in BT	0.0015
Figure 1E	Unpaired t test with Welch's correction	BA.2.86 in Con vs BA.2.86 in BT	0.0026
Figure 1E	Unpaired t test with Welch's correction	D614G in Con vs D614G in BT	0.0156
Figure 1E	Unpaired t test with Welch's correction	BA.2 in Con vs BA.2 in BT	0.0105
Figure 1E	Unpaired t test with Welch's correction	BA.1 in Con vs BA.1 in BT	0.0109
Figure 1E	Unpaired t test with Welch's correction	Delta in Con vs Delta in BT	0.012
Figure 1F	Unpaired t test	EMCV in Con vs EMCV in BT	0.0025

Figure 1F	Unpaired t test	HSV in Con vs HSV in BT	0.0043
Figure 1F	Unpaired t test	IAV in Con vs IAV in BT	0.0018
Figure 1F	Unpaired t test	VSV in Con vs VSV in BT	0.0031
Figure 1F	Unpaired t test	BA.2.86 in Con vs BA.2.86 in BT	<0.0001
Figure 1F	Unpaired t test with Welch's correction	D614G in Con vs D614G in BT	0.0032
Figure 1F	Unpaired t test	BA.2 in Con vs BA.2 in BT	<0.0001
Figure 1F	Unpaired t test with Welch's correction	BA.1 in Con vs BA.1 in BT	0.0045
Figure 1F	Unpaired t test	Delta in Con vs Delta in BT	<0.0001
Figure 1G	Log-rank (Mantel-Cox) test	Con vs BT	0.0018
Figure 1G	Unpaired t test with Welch's correction	Con vs BT (MHV-Blood MHV mRNA)	0.0217
Figure 1G	Unpaired t test	Con vs BT (MHV-Blood Il6 mRNA)	0.0352
Figure 1G	Unpaired t test with Welch's correction	Con vs BT (MHV-Liver MHV mRNA)	0.0431
Figure 1G	Unpaired t test	Con vs BT (MHV-Liver Il6 mRNA)	0.0417
Figure 1H	Log-rank (Mantel-Cox) test	Con vs BT	0.0034
Figure 1H	Unpaired t test with Welch's correction	Con vs BT (VSV-Blood VSV mRNA)	0.027
Figure 1H	Unpaired t test	Con vs BT (VSV-Blood Il6 mRNA)	0.005
Figure 1H	Mann Whitney test	Con vs BT (VSV-Liver VSV mRNA)	0.003
Figure 1H	Mann Whitney test	Con vs BT (VSV-Liver Il6 mRNA)	0.0005
Figure 1I	Log-rank (Mantel-Cox) test	Con vs BT	0.0153
Figure 1I	Mann Whitney test	Con vs BT (HSV-Blood HSV mRNA)	0.008
Figure 1I	Mann Whitney test	Con vs BT (HSV-Blood Il6 mRNA)	0.0043
Figure 1I	Mann Whitney test	Con vs BT (HSV-Liver HSV mRNA)	0.012

Figure 1I	Unpaired t test with Welch's correction	Con vs BT (HSV-Liver Il6 mRNA)	0.04
Figure 1J	Log-rank (Mantel-Cox) test	Con vs BT	0.0372
Figure 1J	Mann Whitney test	Con vs BT (IAV-Blood IAV mRNA)	0.0022
Figure 1J	Mann Whitney test	Con vs BT (IAV-Blood Il6 mRNA)	0.0022
Figure 1J	Unpaired t test with Welch's correction	Con vs BT (IAV-Lung IAV mRNA)	0.0415
Figure 1J	Mann Whitney test	Con vs BT (IAV-Lung Il6 mRNA)	0.0022
Figure 1K	Log-rank (Mantel-Cox) test	Con vs BT	0.0671
Figure 1K	Mann Whitney test	Con vs BT (EMCV-Blood EMCV mRNA)	0.019
Figure 1K	Unpaired t test	Con vs BT (EMCV-Blood Il6 mRNA)	0.0266
Figure 1K	Unpaired t test	Con vs BT (EMCV-Liver EMCV mRNA)	0.0289
Figure 1K	Mann Whitney test	Con vs BT (EMCV-Liver Il6 mRNA)	>0.9999
Figure 2D	One-way ANOVA	DMSO vs 100μM (Ifnb1 mRNA)	<0.0001
Figure 2D	One-way ANOVA	DMSO vs 100μM (Isg15 mRNA)	0.0006
Figure 2E	One-way ANOVA	DMSO vs 100μM (RAW 264.7)	<0.0001
Figure 2E	One-way ANOVA	DMSO vs 100μM (HT1080)	<0.0001
Figure 2E	One-way ANOVA	DMSO vs 100μM (HT29)	0.0014
Figure 2E	One-way ANOVA	DMSO vs 100μM (HeLa)	<0.0001
Figure 2F	One-way ANOVA	Con vs 100μM (IFNB1 mRNA)	<0.0001
Figure 2F	One-way ANOVA	Con vs 100μM (OAS2 mRNA)	0.0001
Figure 2G	Unpaired t test	Con vs BT (Blood Ifnb1 mRNA)	0.0006
Figure 2G	Unpaired t test	Con vs BT (Heart Ifnb1 mRNA)	0.032
Figure 2G	Unpaired t test	Con vs BT (Liver Ifnb1 mRNA)	0.0281

Figure 2G	Unpaired t test	Con vs BT (Spleen Ifnb1 mRNA)	0.0036
Figure 2G	Unpaired t test	Con vs BT (Lung Ifnb1 mRNA)	0.001
Figure 2G	Unpaired t test	Con vs BT (Kidney Ifnb1 mRNA)	0.0046
Figure 2G	Unpaired t test	Con vs BT (Blood Oas2 mRNA)	0.0383
Figure 2G	Unpaired t test	Con vs BT (Heart Oas2 mRNA)	0.0051
Figure 2G	Unpaired t test	Con vs BT (Liver Oas2 mRNA)	0.0121
Figure 2G	Unpaired t test	Con vs BT (Spleen Oas2 mRNA)	0.001
Figure 2G	Unpaired t test	Con vs BT (Lung Oas2 mRNA)	0.9304
Figure 2G	Unpaired t test	Con vs BT (Kidney Oas2 mRNA)	0.0174
Figure 2H	Two-way ANOVA	DMSO vs 100μM (WT)	<0.0001
Figure 2H	Two-way ANOVA	DMSO vs 100μM (TBK1$^{-/-}$)	>0.9999
Figure 2J	Two-way ANOVA	DMSO vs 100μM (WT Ifnb1 mRNA)	<0.0001
Figure 2J	Two-way ANOVA	DMSO vs 100μM (IRF3$^{-/-}$ Ifnb1 mRNA)	>0.9999
Figure 2J	Two-way ANOVA	DMSO vs 100μM (WT Type I IFN)	<0.0001
Figure 2J	Two-way ANOVA	DMSO vs 100μM (IRF3$^{-/-}$ Type I IFN)	0.2091
Figure 2K	Two-way ANOVA	DMSO vs 100μM (WT)	<0.0001
Figure 2K	Two-way ANOVA	DMSO vs 100μM (IRF3$^{-/-}$)	0.062
Figure 3D	Two-way ANOVA	DMSO vs 100μM (WT)	<0.0001
Figure 3D	Two-way ANOVA	WT-100μM vs cGAS$^{-/-}$-100μM	<0.0001
Figure 3D	Two-way ANOVA	WT-100μM vs STING$^{-/-}$-100μM	<0.0001
Figure 3E	Two-way ANOVA	DMSO vs 100μM (WT)	<0.0001
Figure 3E	Two-way ANOVA	DMSO vs 100μM (cGAS$^{-/-}$)	0.0226

Figure 3E	Two-way ANOVA	DMSO vs 100μM (STING$^{-/-}$)	0.0114
Figure 3G	Two-way ANOVA	DMSO vs 100μM (WT)	<0.0001
Figure 3G	Two-way ANOVA	DMSO vs 100μM (cGAS$^{-/-}$)	0.0165
Figure 3G	Two-way ANOVA	DMSO vs 100μM (STING$^{-/-}$)	0.0023
Figure 3H	Log-rank (Mantel-Cox) test	Con vs BT (WT)	0.0293
Figure 3H	Log-rank (Mantel-Cox) test	Con vs BT (cGAS$^{-/-}$)	0.5172
Figure 3H	Unpaired t test	Con vs BT (WT)	0.0498
Figure 3H	Unpaired t test	Con vs BT (cGAS$^{-/-}$)	0.7719
Figure 3I	Log-rank (Mantel-Cox) test	Con vs BT (WT)	0.0323
Figure 3I	Log-rank (Mantel-Cox) test	Con vs BT (cGAS$^{-/-}$)	0.7809
Figure 3I	Unpaired t test	Con vs BT (WT)	0.0222
Figure 3I	Unpaired t test	Con vs BT (cGAS$^{-/-}$)	0.7837
Figure 3J	Unpaired t test	DMSO vs BT	0.0002
Figure 3N	One-way ANOVA	DMSO vs 25μM (Without dsDNA)	<0.0001
Figure 3N	One-way ANOVA	DMSO vs 100μM (Without dsDNA)	<0.0001
Figure 3N	One-way ANOVA	DMSO vs 25μM (With dsDNA)	<0.0001
Figure 3N	One-way ANOVA	DMSO vs 100μM (With dsDNA)	<0.0001
Figure 4A	Unpaired t test	Con vs BT (HDAC1)	0.0297
Figure 4A	Unpaired t test	Con vs BT (HDAC2)	0.0155
Figure 4A	Unpaired t test	Con vs BT (HDAC3)	0.0044
Figure 4A	Unpaired t test	Con vs BT (HDAC4)	0.0056
Figure 4A	Unpaired t test	Con vs BT (HDAC5)	0.0156

Figure 4A	Unpaired t test	Con vs BT (HDAC6)	0.0098
Figure 4B	One-way ANOVA	Con vs 100μM	0.0007
Figure 4I	Unpaired t test	H3K27ac vs TRIM28	0.0005
Figure 4J	One-way ANOVA	0 vs 12 (OAS3) (H3K27ac)	0.0011
Figure 4J	One-way ANOVA	0 vs 12 (IFI44) (H3K27ac)	0.0016
Figure 4J	One-way ANOVA	0 vs 12 (OAS3) (Trim28)	0.0128
Figure 4J	One-way ANOVA	0 vs 12 (IFI44) (Trim28)	0.0052
Figure 5E	Two-way ANOVA	DMSO vs 100μM (WT)	<0.0001
Figure 5E	Two-way ANOVA	DMSO vs 100μM ($MDA5^{-/-}$)	0.0558
Figure 5E	Two-way ANOVA	DMSO vs 100μM ($RIG\text{-}I^{-/-}$)	0.2221
Figure 5E	Two-way ANOVA	DMSO vs 100μM ($MAVS^{-/-}$)	0.1399
Figure 5F	Two-way ANOVA	DMSO vs 100μM (WT)	<0.0001
Figure 5F	Two-way ANOVA	DMSO vs 100μM ($MDA5^{-/-}$)	0.0205
Figure 5F	Two-way ANOVA	DMSO vs 100μM ($RIG\text{-}I^{-/-}$)	0.0789
Figure 5F	Two-way ANOVA	DMSO vs 100μM ($MAVS^{-/-}$)	0.0773
Figure 5H	Two-way ANOVA	DMSO vs 100μM (WT)	<0.0001
Figure 5H	Two-way ANOVA	DMSO vs 100μM ($MDA5^{-/-}$)	0.0011
Figure 5H	Two-way ANOVA	DMSO vs 100μM ($RIG\text{-}I^{-/-}$)	0.0029
Figure 5H	Two-way ANOVA	DMSO vs 100μM ($MAVS^{-/-}$)	0.0016
Figure 5I	Log-rank (Mantel-Cox) test	Con vs BT (WT)	0.0293
Figure 5I	Log-rank (Mantel-Cox) test	Con vs BT ($MAVS^{-/-}$)	0.9172
Figure 5I	Unpaired t test	Con vs BT (WT)	0.0498

Figure 5I	Unpaired t test	Con vs BT ($MAVS^{-/-}$)	0.576
Figure 5J	Log-rank (Mantel-Cox) test	Con vs BT (WT)	0.0323
Figure 5J	Log-rank (Mantel-Cox) test	Con vs BT ($MAVS^{-/-}$)	0.7121
Figure 5J	Unpaired t test	Con vs BT (WT)	0.0222
Figure 5J	Unpaired t test	Con vs BT ($MAVS^{-/-}$)	0.9582
Figure 5K	Unpaired t test	Con vs BT (RIGI-IP-qPCR)	0.0346
Figure 5K	Unpaired t test	Con vs BT (MDA5-IP-qPCR)	0.0094
Figure 5L	Two-way ANOVA	DMSO vs 100μM (WT Type I IFN)	<0.0001
Figure 5L	Two-way ANOVA	DMSO vs 100μM ($MAVS/cGAS^{-/-}$ Type I IFN)	0.9481
Figure 5L	Two-way ANOVA	DMSO vs 100μM (WT IFNB1 mRNA)	<0.0001
Figure 5L	Two-way ANOVA	DMSO vs 100μM ($MAVS/cGAS^{-/-}$ IFNB1 mRNA)	>0.9999
Figure 5M	Two-way ANOVA	DMSO vs 100μM (WT)	<0.0001
Figure 5M	Two-way ANOVA	DMSO vs 100μM ($MAVS/cGAS^{-/-}$)	0.8855
