# Supplementary material for: Betrixaban activates cGAS and ERVs to promote dual nucleic-sensing antiviral immunity
Source: EMBO Mol Med. 2026 Mar 23;18(5):1563–91. doi: 10.1038/s44321-025-00356-7 (PMC13179341; doi:10.1038/s44321-025-00356-7)
Supplement: Supplementary file 5 — Source data Fig. 1 [file 44321_2025_356_MOESM5_ESM.zip › Figure1/1C/1C_WB picture.pptx]

## Slide 1
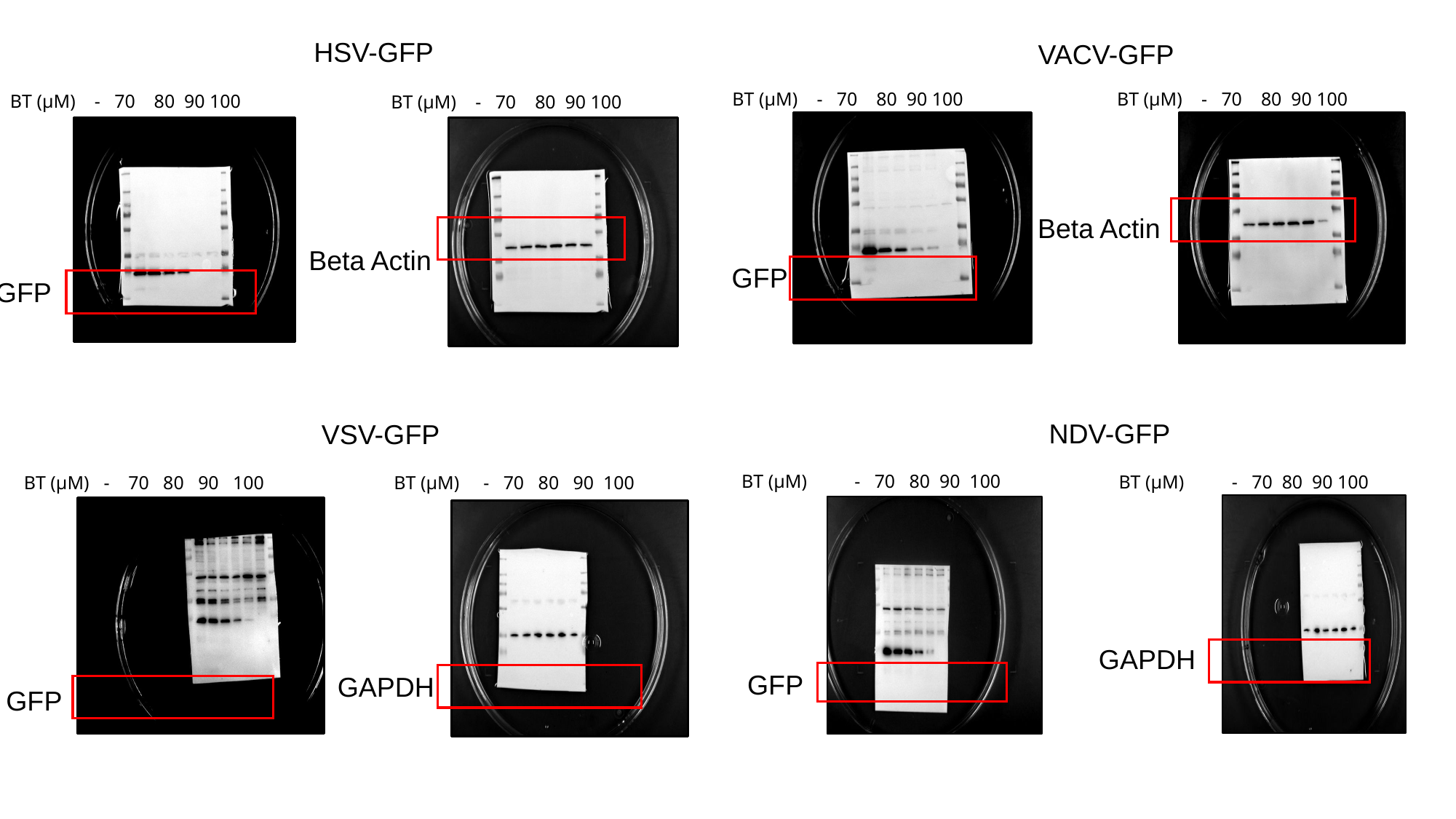

HSV-GFP
VACV-GFP
BT (μM)
 - 70 80 90 100
BT (μM)
 - 70 80 90 100
BT (μM)
 - 70 80 90 100
BT (μM)
 - 70 80 90 100
Beta Actin
Beta Actin
GFP
GFP
NDV-GFP
VSV-GFP
BT (μM)
 - 70 80 90 100
BT (μM)
 - 70 80 90 100
BT (μM)
BT (μM)
 - 70 80 90 100
 - 70 80 90 100
GAPDH
GFP
GAPDH
GFP
