# Supplementary material for: Betrixaban activates cGAS and ERVs to promote dual nucleic-sensing antiviral immunity
Source: EMBO Mol Med. 2026 Mar 23;18(5):1563–91. doi: 10.1038/s44321-025-00356-7 (PMC13179341; doi:10.1038/s44321-025-00356-7)
Supplement: Supplementary file 6 — Source data Fig. 2 [file 44321_2025_356_MOESM6_ESM.zip › Figure2/2D/2E_HT29 WB picture.pptx]

## Slide 1
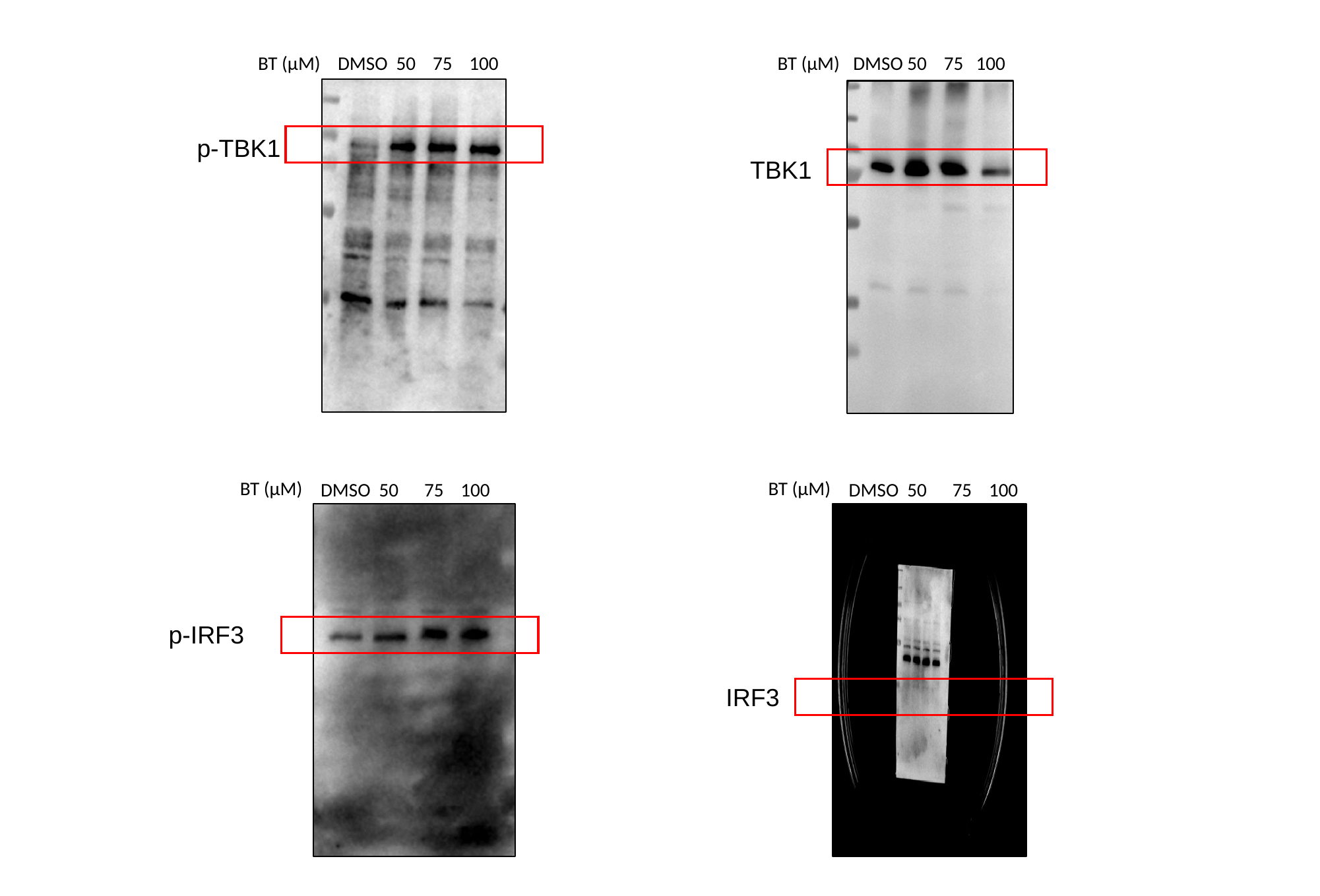

BT (μM)
 DMSO 50 75 100
BT (μM)
DMSO 50 75 100
p-TBK1
TBK1
BT (μM)
BT (μM)
 DMSO 50 75 100
 DMSO 50 75 100
p-IRF3
IRF3

## Slide 2
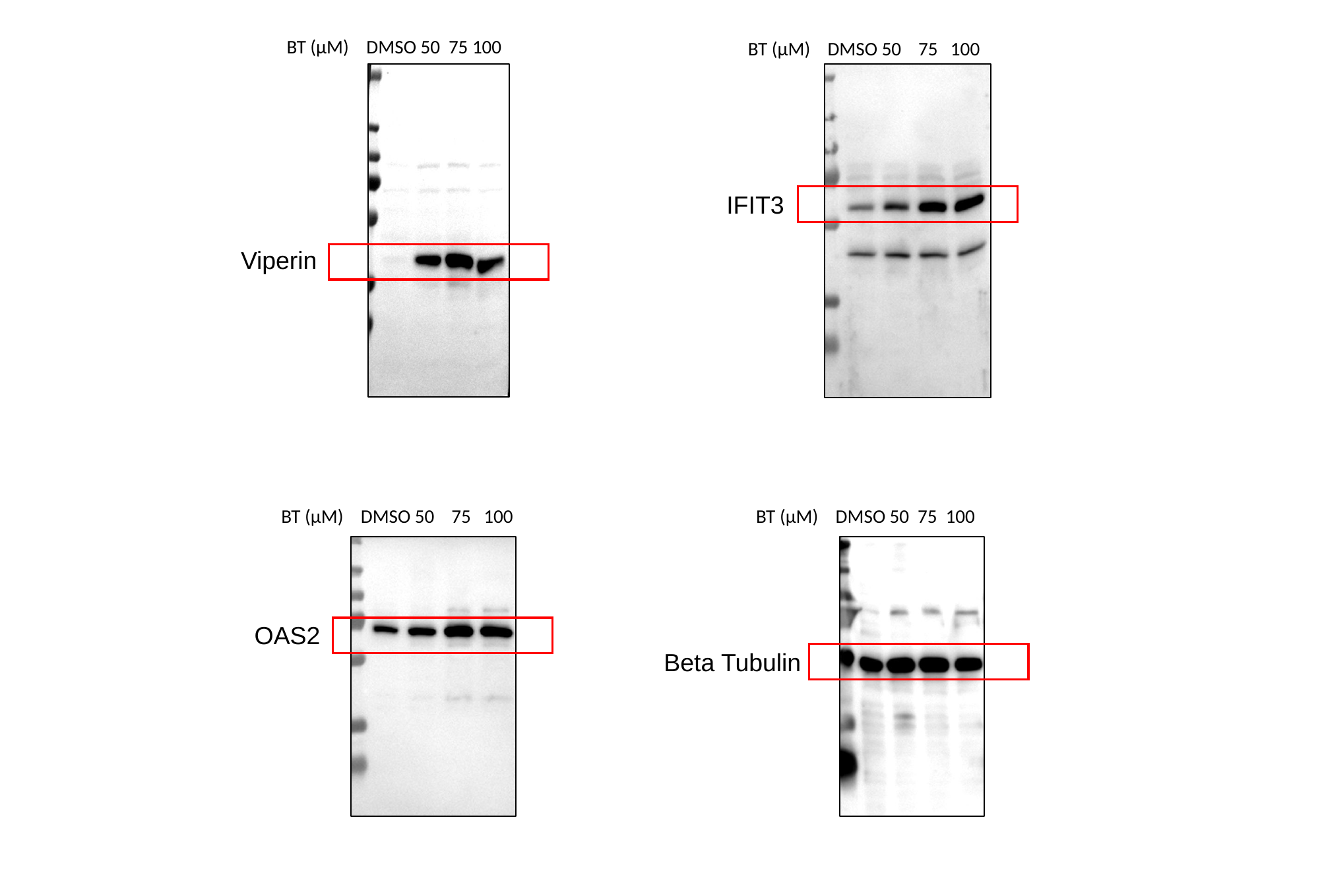

BT (μM)
DMSO 50 75 100
BT (μM)
DMSO 50 75 100
IFIT3
Viperin
BT (μM)
DMSO 50 75 100
BT (μM)
DMSO 50 75 100
OAS2
Beta Tubulin
