# Supplementary material for: Betrixaban activates cGAS and ERVs to promote dual nucleic-sensing antiviral immunity
Source: EMBO Mol Med. 2026 Mar 23;18(5):1563–91. doi: 10.1038/s44321-025-00356-7 (PMC13179341; doi:10.1038/s44321-025-00356-7)
Supplement: Supplementary file 6 — Source data Fig. 2 [file 44321_2025_356_MOESM6_ESM.zip › Figure2/2H/2H_WB picture.pptx]

## Slide 1
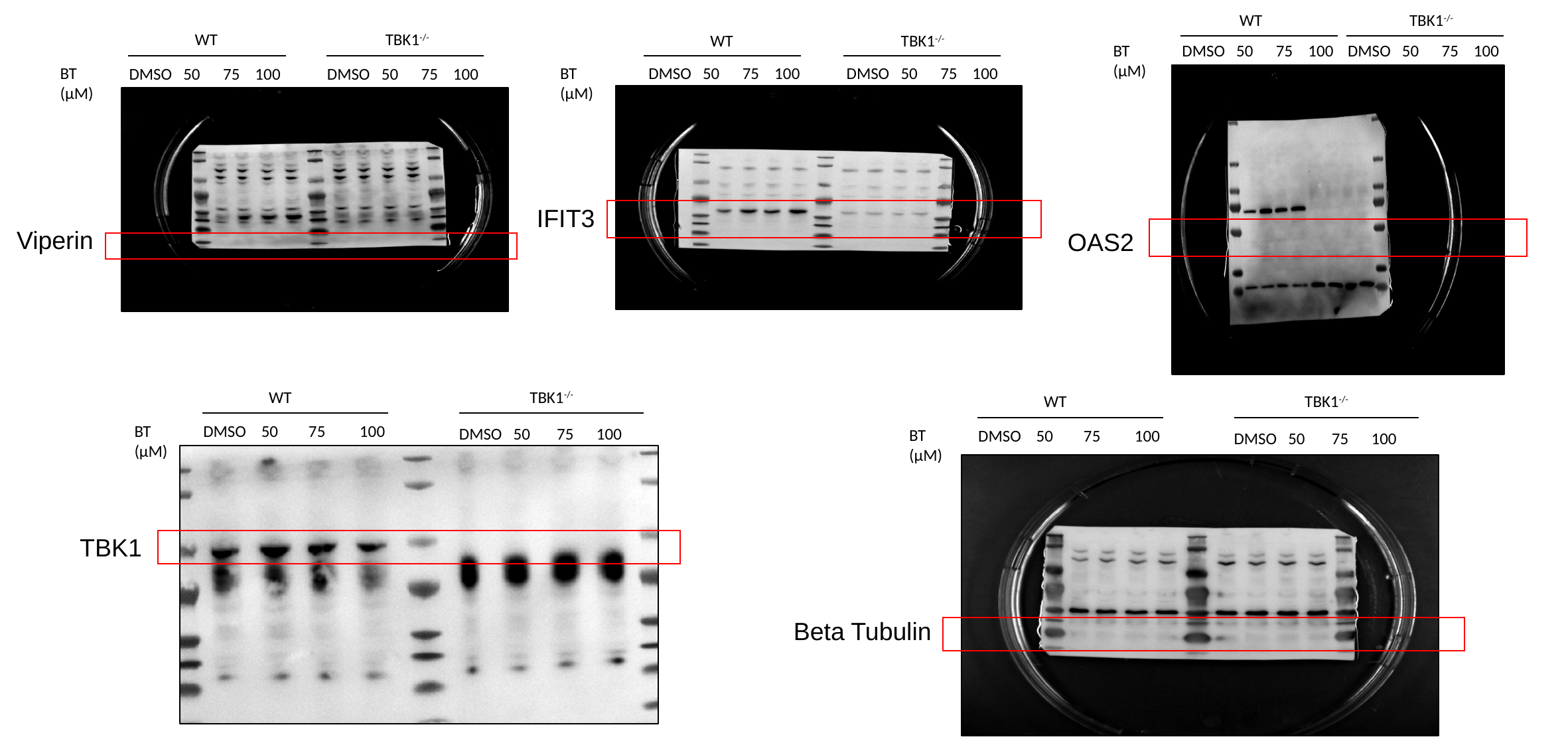

WT
TBK1-/-
TBK1-/-
WT
TBK1-/-
WT
BT (μM)
DMSO 50 75 100
DMSO 50 75 100
BT (μM)
BT (μM)
DMSO 50 75 100
DMSO 50 75 100
DMSO 50 75 100
DMSO 50 75 100
IFIT3
Viperin
OAS2
TBK1-/-
WT
TBK1-/-
WT
BT (μM)
DMSO 50 75 100
DMSO 50 75 100
BT (μM)
DMSO 50 75 100
DMSO 50 75 100
TBK1
Beta Tubulin
