# Supplementary figures and images for: Betrixaban activates cGAS and ERVs to promote dual nucleic-sensing antiviral immunity
Source: EMBO Mol Med. 2026 Mar 23;18(5):1563–91. doi: 10.1038/s44321-025-00356-7 (PMC13179341; doi:10.1038/s44321-025-00356-7)

## Slide 1
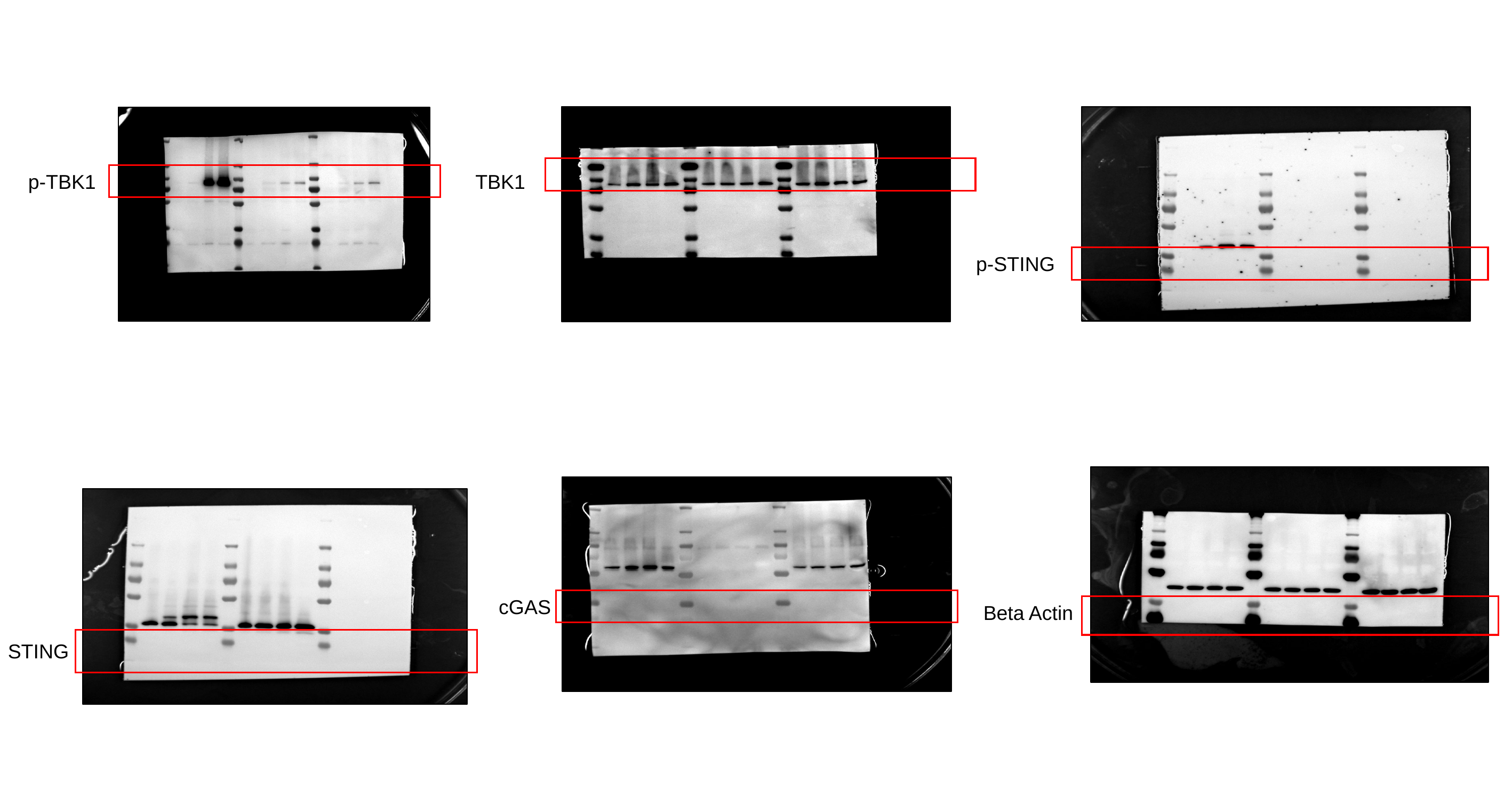

p-TBK1
TBK1
p-STING
cGAS
Beta Actin
STING

Supplement: Supplementary file 7 — Source data Fig. 3 [file 44321_2025_356_MOESM7_ESM.zip › Figure3/3F/3F_WB picture.pptx]

## Slide 1
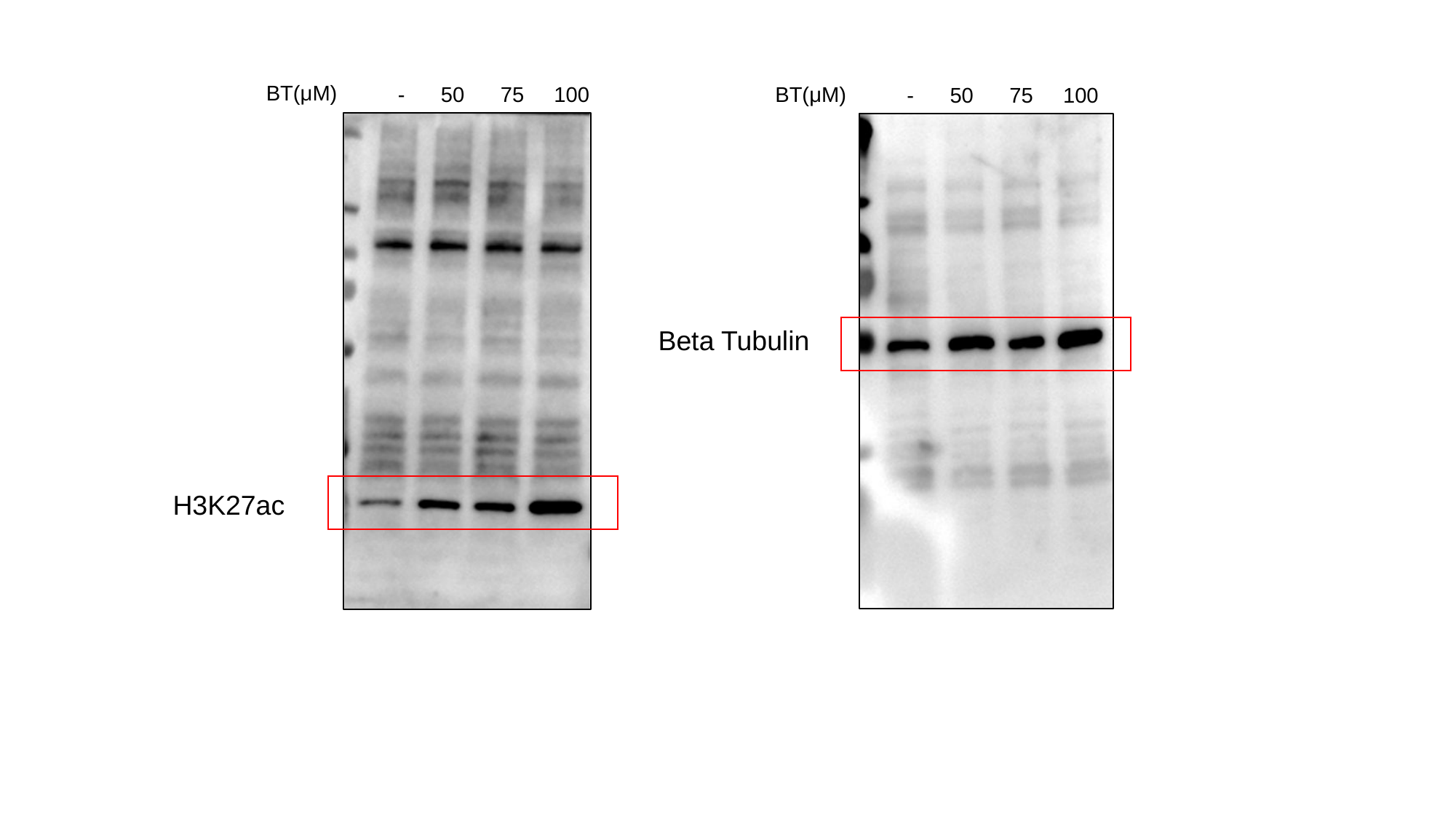

BT(μM)
BT(μM)
- 50 75 100
- 50 75 100
Beta Tubulin
H3K27ac

Supplement: Supplementary file 8 — Source data Fig. 4 [file 44321_2025_356_MOESM8_ESM.zip › Figure4/4C/4C_WB picture.pptx]

## Slide 1
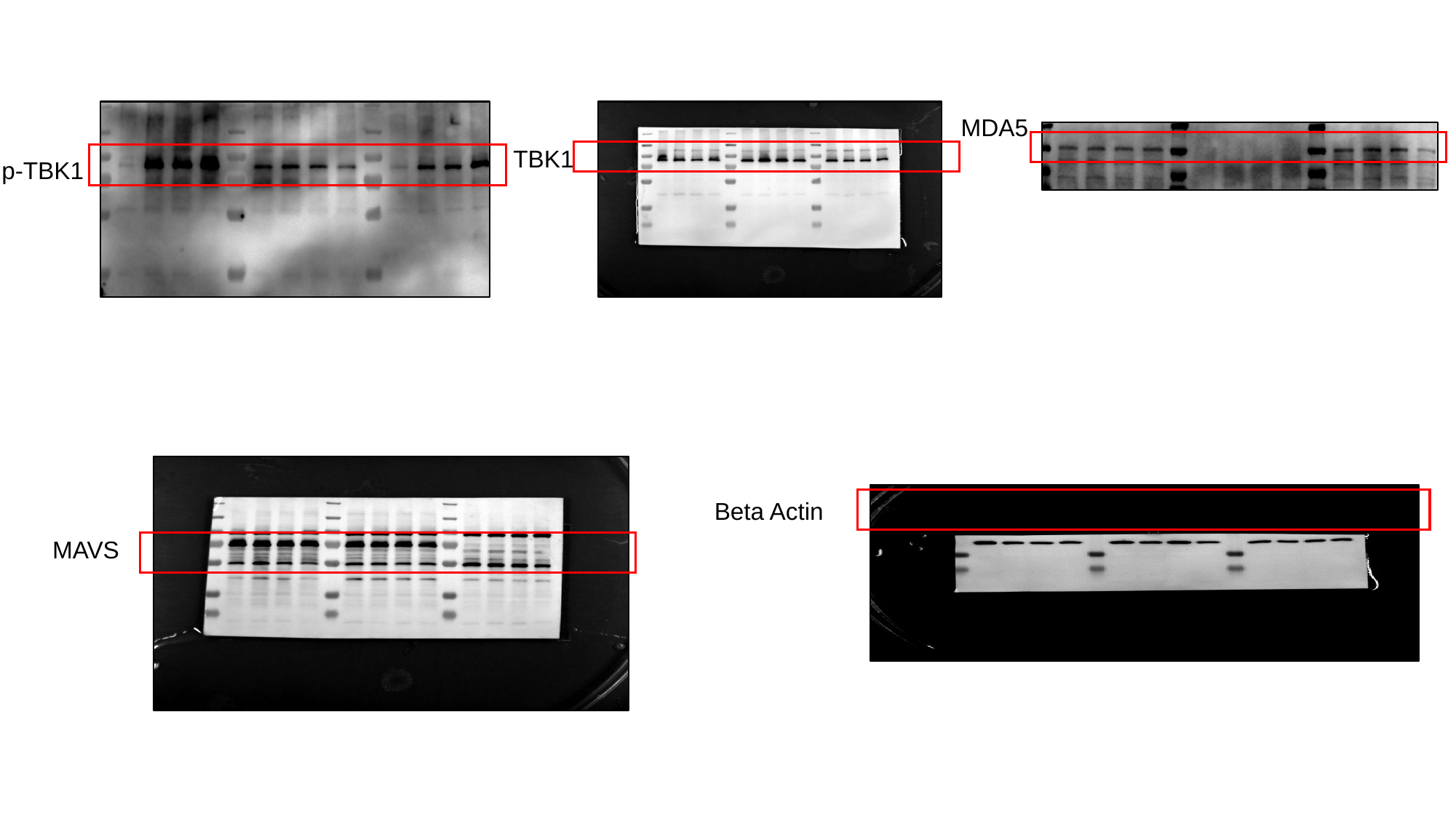

MDA5
TBK1
p-TBK1
Beta Actin
MAVS

## Slide 2
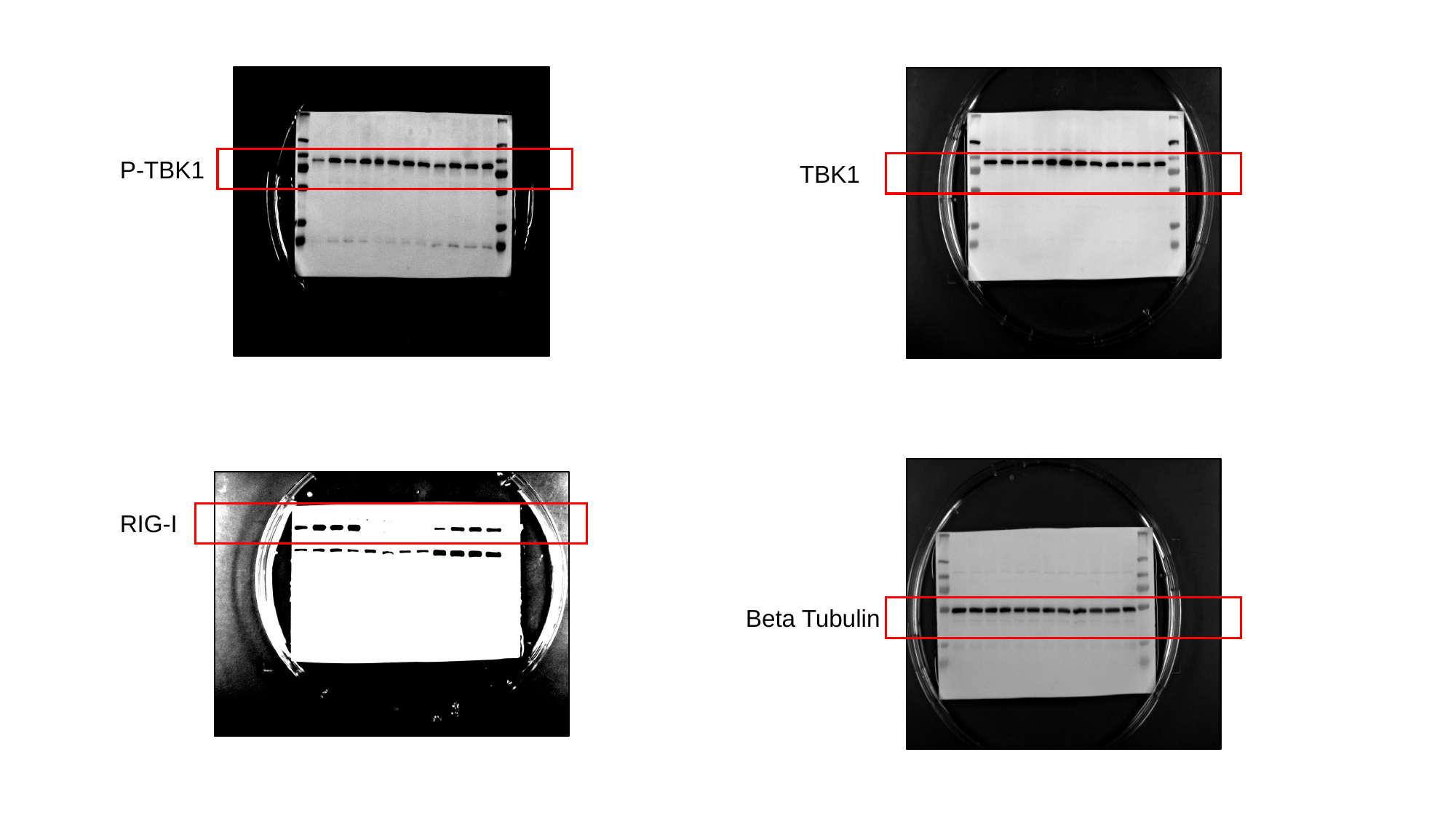

P-TBK1
TBK1
RIG-I
Beta Tubulin

Supplement: Supplementary file 9 — Source data Fig. 5 [file 44321_2025_356_MOESM9_ESM.zip › Figure5/5G/5G_WB picture.pptx]
