# Supplementary material for: Betrixaban activates cGAS and ERVs to promote dual nucleic-sensing antiviral immunity
Source: EMBO Mol Med. 2026 Mar 23;18(5):1563–91. doi: 10.1038/s44321-025-00356-7 (PMC13179341; doi:10.1038/s44321-025-00356-7)
Supplement: Supplementary file 11 — Expanded View Figures [file 44321_2025_356_MOESM11_ESM.pdf]

Expanded View Figures

Figure EV1. Betrixaban (BT) establishes a host-protective antiviral state in vitro and in vivo.

(**A**) Representative density contour plot images of flow cytometry. RAW 264.7 cells treated with DMSO (Con) or 100 μM BT and infected with HSV-GFP, VACV-GFP, VSV-GFP or NDV-GFP (MOI = 0.1). (**B**) RT-qPCR quantification of viral RNA, luciferase to detect BA.2.86, D614G, BA.2, BA.1, Delta and fluorescence (green) and bright-field images in HeLa cells. (**C**, **D**) RT-qPCR quantification of viral RNA, *Il1b, Il6, Tnfa* mRNA treated with DMSO (Con) or 100 μM BT and infected with different viruses for 12 h in BMDMs. (**E**, **F**) RT-qPCR and luciferase to detect *Il1b, Il6, Tnfa* mRNA treated with DMSO (Con) or 100 μM BT and infected with different viruses for 12 h in RAW 264.7 cells. (**G**) Virus titers in RAW264.7 and HT1080 cells infected with viruses following the treatment of BT. (**H**) RT-qPCR of *Il1b*, *Il6* and *Tnfa* in RAW 264.7 cells following BT treatment (50, 75, 100 μM) for 12 h. (**I**) In vivo detection of *Il1b*, *Il6* and *Tnfa* mRNA in blood, heart, liver, lung, spleen and kidney of C57BL/6 J mice 6 h after a single intraperitoneal injection of BT (50 mg/kg). (**J**) Time-of-addition assay. After VSV or HSV-1 infection (37 °C 1 h; washes), BT (50, 75, 100 μM) was added at 0, +1, +2, +4, or +6 h post-infection. We quantified viruses mRNA using RT-qPCR. (**K**) 4 °C attachment assay. Cells were pre-chilled and exposed to VSV or HSV-1 at 4 °C for 1 h, followed by ice-cold washes. Viral genomes bound to the cell surface were quantified by RT-qPCR. (**L**) 37 °C internalization assay. After 4 °C binding and washes, cells were shifted to 37 °C for 60 min to allow uptake, then subjected to a brief trypsin wash to remove surface-bound virions. Intracellular viral RNA was measured by RT-qPCR. (**M**) Cell viability measured by CCK-8 assay in RAW 264.7, HeLa, HT1080, HT29, PBMCs and BMDMs (top). *IFNB1* (RT-qPCR) and VSV-GFP (%GFP, flow cytometry) dose-responses in RAW264.7 cells, EC50/IC50 indicated (bottom). (**N**) Virus titers in the livers of mice infected with a lethal dose of VSV, HSV-1 with or without the treatment of BT (as described in the "Methods"). (**O–Q**) Mice received daily intraperitoneal injections for 14 consecutive days of either no treatment (MOCK), solvent alone (PBS), or the test compound (BT, 50 mg/kg/day). At the end of the dosing period, major organs were harvested for analysis. Body weight and organ weights (heart, liver, spleen, lung, kidney) (**O**), representative photographs of dissected organs (**P**), histological examination of organs. Scale bars, 100 μm (×20) (**Q**). Data are shown as mean ± SEM. N.S., not significant, $P > 0.05$; *$P < 0.05$; **$P < 0.01$; ***$P < 0.001$.

© The Author(s)

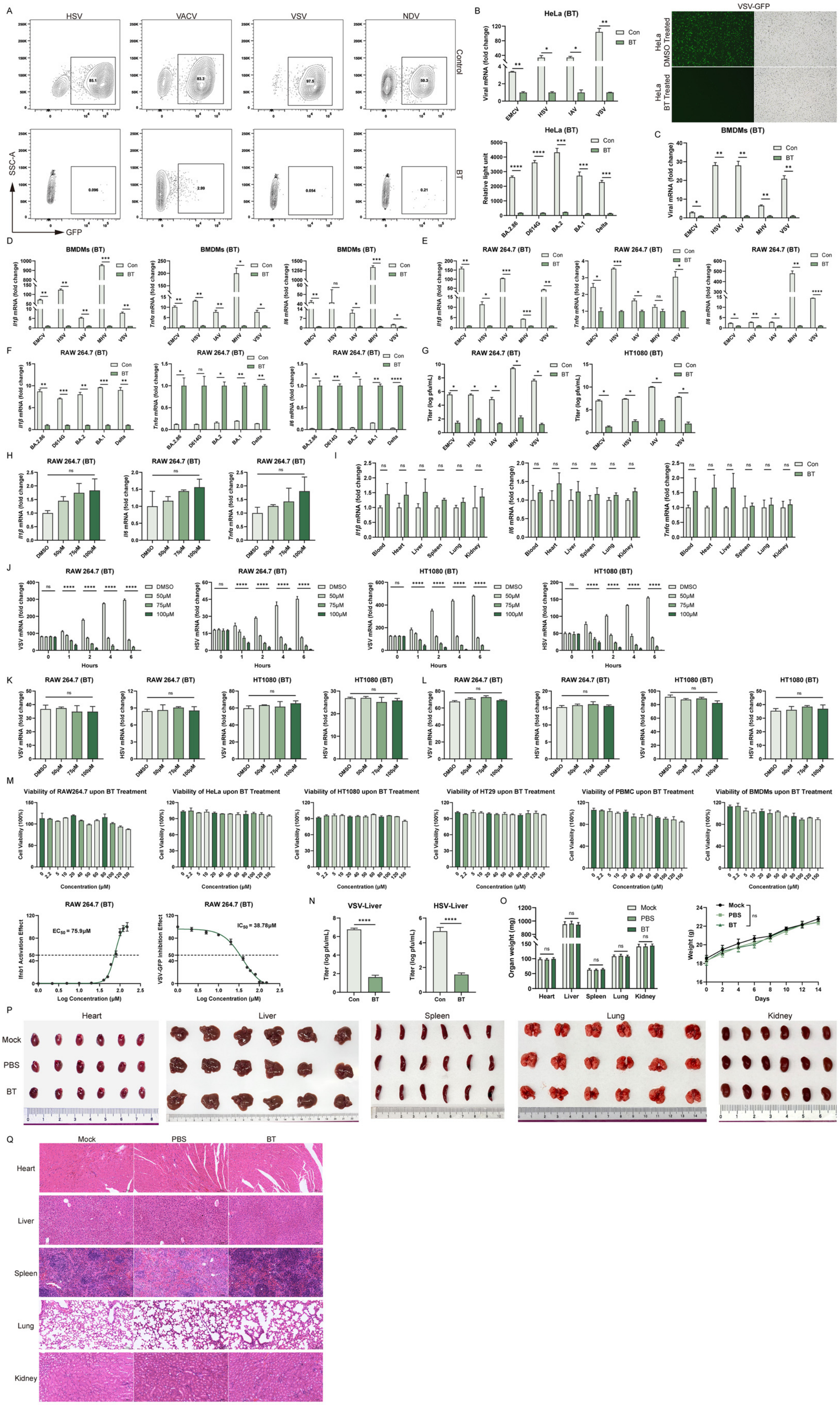
A
HSV
VACV
VSV
NDV
Control
BT
SSC-A
GFP
B
HeLa (BT)
Viral mRNA (fold change)
Con
BT
EMCV
HSV
IAV
VSV
VSV-GFP
HeLa DMSO Treated
HeLa BT Treated
Relative light unit
BA.2.86
D614G
BA.2
BA.1
Delta
C
BMDMs (BT)
MHV
D
E
RAW 264.7 (BT)
F
G
HT1080 (BT)
Titer (log pfu/mL)
H
I
Blood
Heart
Liver
Spleen
Lung
Kidney
J
DMSO
50μM
75μM
100μM
Hours
K
L
M
Viability of RAW264.7 upon BT Treatment
Viability of HeLa upon BT Treatment
Viability of HT1080 upon BT Treatment
Viability of HT29 upon BT Treatment
Viability of PBMC upon BT Treatment
Viability of BMDMs upon BT Treatment
Cell Viability (100%)
Concentration (μM)
Log Concentration (μM)
N
VSV-Liver
HSV-Liver
O
Mock
PBS
Organ weight (mg)
Weight (g)
Days
P
Q

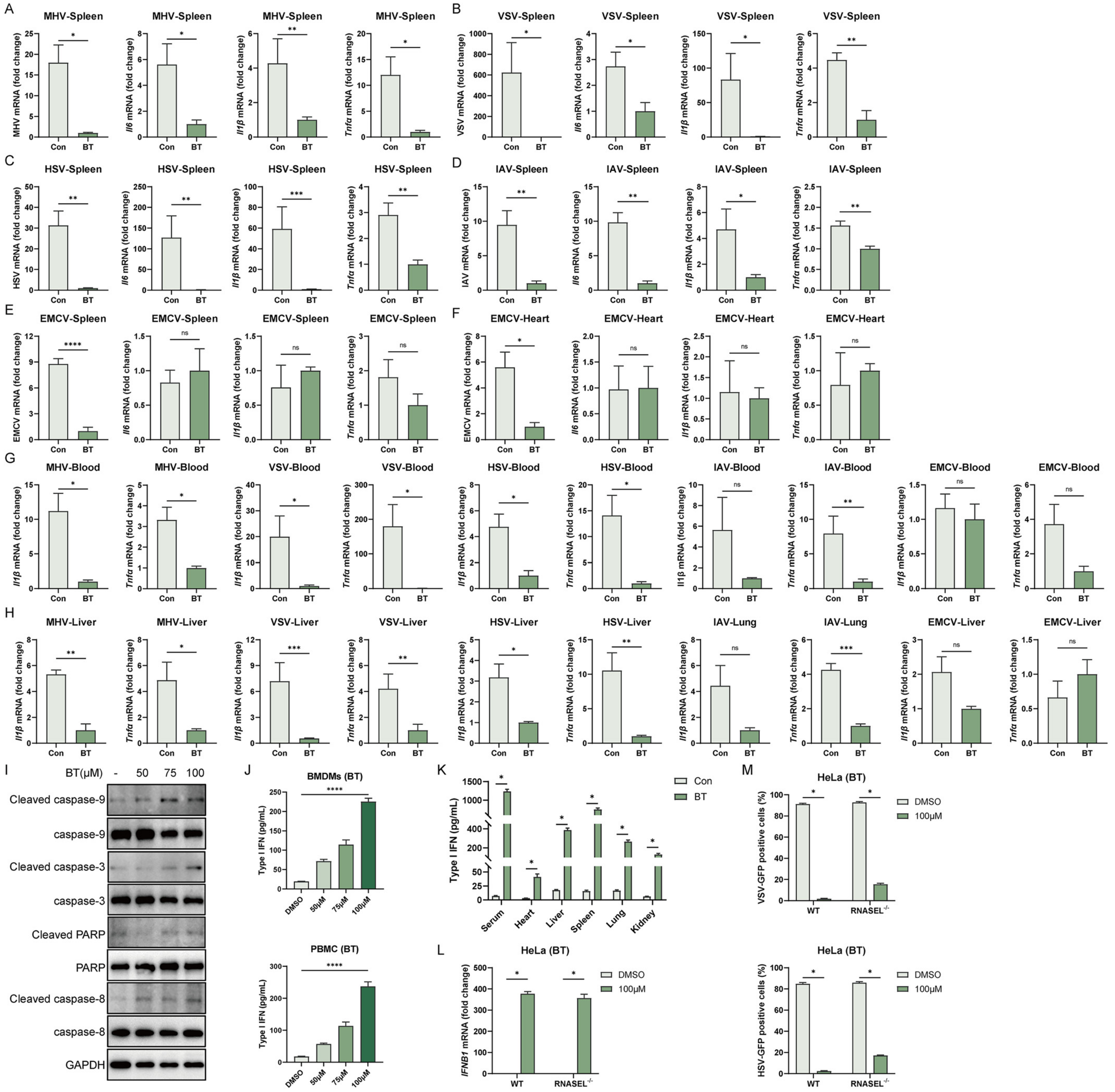

Figure EV2. Betrixaban (BT) establishes a host-protective antiviral state in vivo.

(**A–H**) BT protected C57BL/6 J mice from lethal viral challenge. Mice infected intraperitoneally with MHV ($n = 10$), VSV ($n = 12$), HSV-1 ($n = 11$), IAV ($n = 13$) or EMCV ($n = 13$) and treated daily with vehicle or BT (50 mg/kg). RT-qPCR quantification of viral RNA and *Il-6, Il1b, Tnfa* mRNA in target organs harvested 24 h post-infection. (**I**) Western blot of total and cleaved caspase-3, total and cleaved PARP, total and cleaved caspase-9, and total and cleaved caspase-8. GAPDH served as a control, the samples were same to Fig. 2D. (**J**) Secreted type I IFN measured by ELISA in the same BMDMs and PBMCs and treatments as in Fig. 2E,F. (**K**) Secreted type I IFN measured by ELISA in mice serum and targeted organs. Six-week-old C57BL/6J mice 6 h after a single intraperitoneal dose of BT (50 mg/kg). (**L**) RT-qPCR quantification of *IFNB1* mRNA in HeLa cells treated with indicated concentrations of BT for 12 h. (**M**) HeLa cells treated with DMSO (Con) or 100 μM BT and infected with HSV-GFP or VSV-GFP (MOI = 0.1), measured by flow cytometry. Data are shown as mean ± SEM. N.S., not significant, $P > 0.05$; $^{*}P < 0.05$; $^{**}P < 0.01$; $^{***}P < 0.001$; $^{****}P < 0.0001$. Western blot quantifications are presented in Appendix Fig. S2.

 © The Author(s)

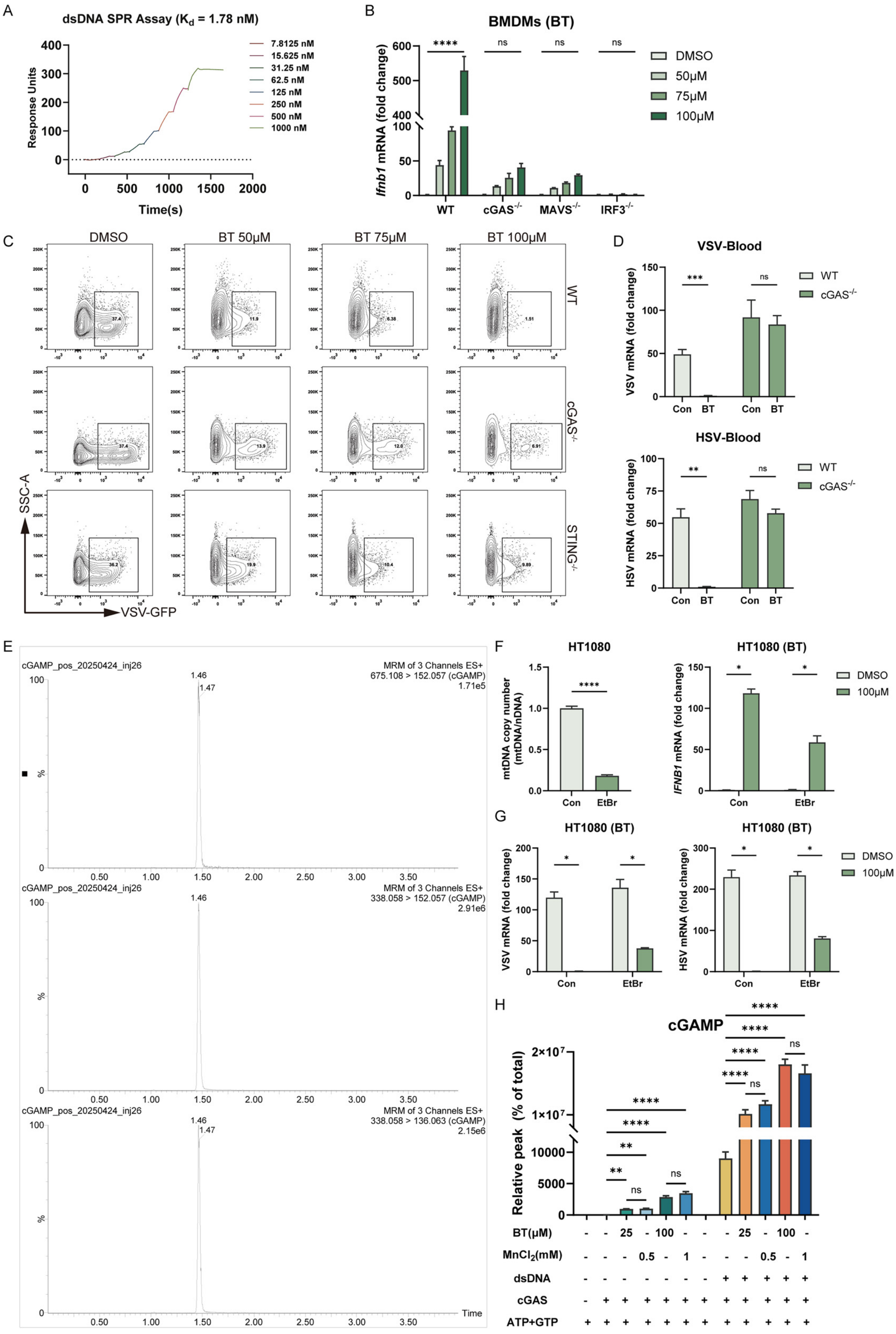
A
dsDNA SPR Assay (Kd = 1.78 nM)
Response Units
Time(s)
B
BMDMs (BT)
Ifnb1 mRNA (fold change)
DMSO
50μM
75μM
100μM
WT
cGAS-/-
MAVS-/-
IRF3-/-
C
DMSO
BT 50μM
BT 75μM
BT 100μM
WT
cGAS-/-
STING-/-
SSC-A
VSV-GFP
D
VSV-Blood
VSV mRNA (fold change)
HSV-Blood
HSV mRNA (fold change)
Con
BT
E
cGAMP_pos_20250424_inj26
MRM of 3 Channels ES+
675.108 > 152.057 (cGAMP)
338.058 > 152.057 (cGAMP)
338.058 > 136.063 (cGAMP)
Time
F
HT1080
mtDNA copy number (mtDNA/nDNA)
HT1080 (BT)
IFNB1 mRNA (fold change)
EtBr
G
VSV mRNA (fold change)
HSV mRNA (fold change)
H
cGAMP
Relative peak (% of total)
BT(μM)
MnCl2(mM)
dsDNA
cGAS
ATP+GTP

Figure EV3. Betrixaban directly binds and sensitizes cGAS activation.

(**A**) SPR sensorgrams of salmon sperm double-stranded DNA binding to recombinant human cGAS at the indicated concentrations. (**B**) RT-qPCR of *Ifnb* in BMDMs from WT, $cGAS^{-/-}$, $MAVS^{-/-}$, and $IRF3^{-/-}$ mice following 100 μM BT treatment for 12 h. (**C**) Representative density contour plot images of flow cytometry of VSV-GFP infection in WT, cGAS knockout and STING knockout HT1080 cells treated with BT (50, 75, 100 μM) and infected (MOI = 0.1) for 12 h. (**D**) Viruses RNA levels in blood measured by RT-qPCR, the treatment is the same to Fig. 3H,I. (**E**) LC-MS/MS characterization of cGAMP standard and monitored ion transitions. (**F**) HT1080 cells were cultured with or without 100 ng/ml ethidium bromide for 6 days. Depletion of mtDNA was measured by Quantitative PCR of mtDNA versus genomic DNA (left). Cells were treated with 100 μM BT for 12 h, *IFNB* mRNA level were monitored by RT-qPCR (right). (**G**) After the depletion of mtDNA, cells were infected with VSV or HSV following the treatment of 100 μM BT or DMSO, RT-qPCR quantification of viral RNA 12 h post-infection. (**H**) In vitro cGAS enzymatic assays: LC-MS quantification of cGAMP production by recombinant cGAS incubated with BT or Mn^{2+}. Data are shown as mean ± SEM. N.S., not significant, $P > 0.05$; $*P < 0.05$; $**P < 0.01$; $***P < 0.001$; $****P < 0.0001$.

 © The Author(s)

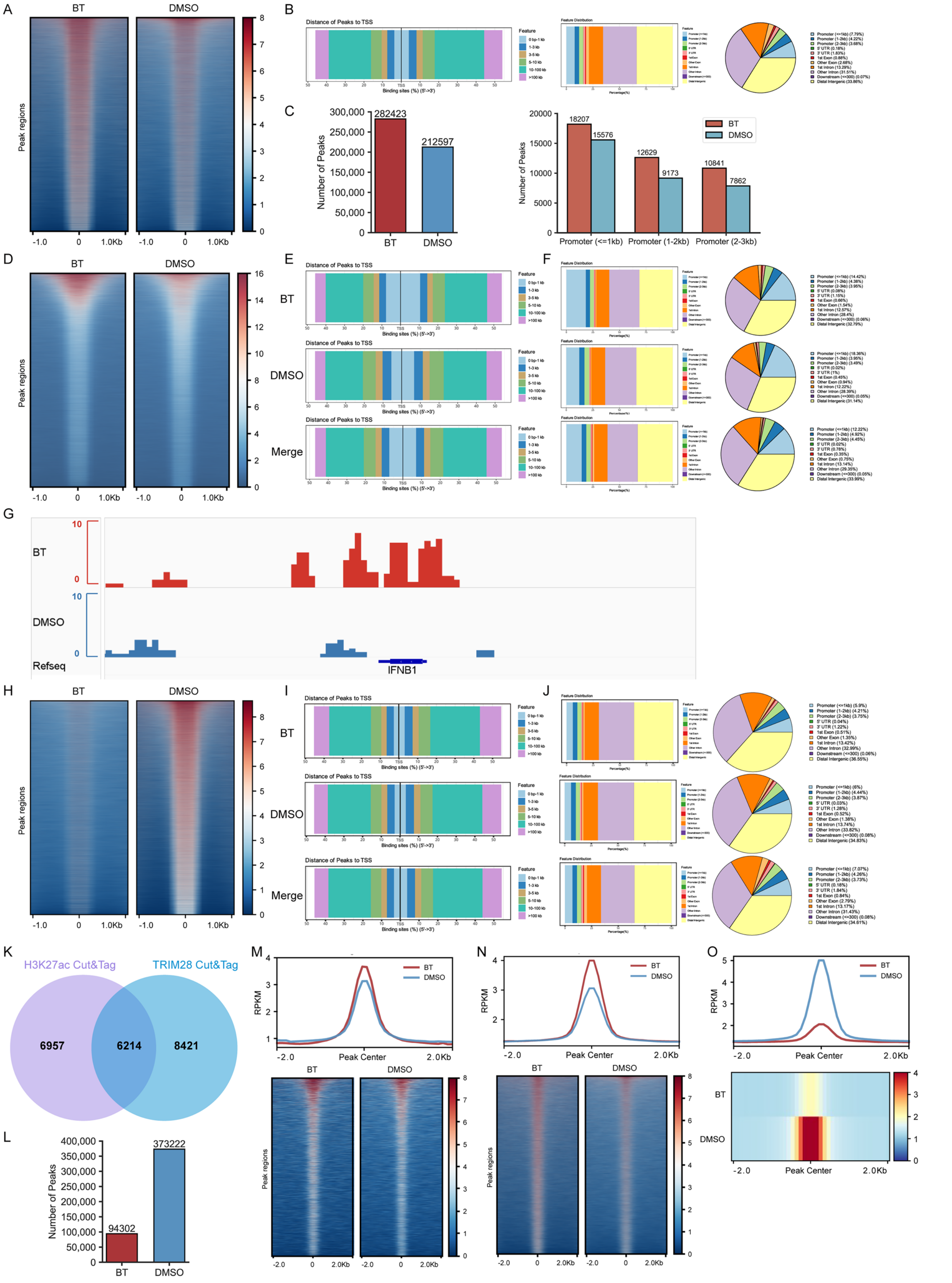
A
BT
DMSO
Peak regions
B
Distance of Peaks to TSS
Feature Distribution
C
Number of Peaks
282423
212597
BT
DMSO
18207
15576
12629
9173
10841
7862
Promoter (<=1kb)
Promoter (1-2kb)
Promoter (2-3kb)
D
E
F
BT
DMSO
Merge
G
Refseq
IFNB1
H
I
J
K
H3K27ac Cut&Tag
TRIM28 Cut&Tag
6957
6214
8421
L
94302
373222
M
N
O
RPKM
Peak Center

Figure EV4. Betrixaban remodels chromatin and reactivates ERVs.

(**A**) Heatmaps of H3K27ac CUT&Tag signal intensity (RPKM) ± 1 kb around all called H3K27ac peak centers in BT- versus DMSO-treated cells. (**B**) Genomic annotation of H3K27ac peaks. distance to the nearest transcription start site (left), bar plot of feature distribution (middle), and pie chart summarizing the proportion of peaks in each genomic feature (right). (**C**) Total number of H3K27ac peaks called in BT and DMSO (left), and number of H3K27ac peaks mapping to promoter regions stratified by distance from the TSS (≤ 1 kb, 1–2 kb, 2–3 kb; right). (**D**) Heatmaps of ATAC-seq signal intensity ±1 kb around all ATAC peak centers in BT- versus DMSO-treated cells. (**E**) Distance-to-TSS annotation for ATAC-seq peaks in BT, DMSO and merged samples. (**F**) Genomic feature distribution of ATAC peaks in BT, DMSO and merged datasets, shown as bar plots and pie charts. (**G**) Genome browser (IGV) snapshots at IFNB1 loci showing H3K27ac CUT&Tag. (**H**) Heatmaps of TRIM28 CUT&Tag signal intensity ±1 kb around all TRIM28 peak centers in BT- versus DMSO-treated cells. (**I**) Distance-to-TSS annotation for TRIM28 peaks in BT (top), DMSO (middle) and merged samples (bottom). (**J**) Genomic feature distribution of TRIM28 peaks in BT, DMSO and merged datasets, shown as bar plots and corresponding pie charts. (**K**) Venn diagram showing overlap between H3K27ac gain and TRIM28 loss at ERV loci upregulated by BT. (**L**) Total number of ATAC-seq peaks called in BT versus DMSO samples at ERV loci. (**M**) Meta-profile (top) and heatmap (bottom) of H3K27ac CUT&Tag signal ±2 kb around the centers of ERV loci that were upregulated by BT. (**N**) Meta-profile (top) and heatmap (bottom) of TRIM28 CUT&Tag signal at the same set of BT-reactivated ERV loci, illustrating loss of TRIM28 binding. (**O**) Meta-profile (top) and heatmap (bottom) of ATAC-seq signal at BT-reactivated ERV loci, showing increased chromatin accessibility upon BT treatment.

 © The Author(s)

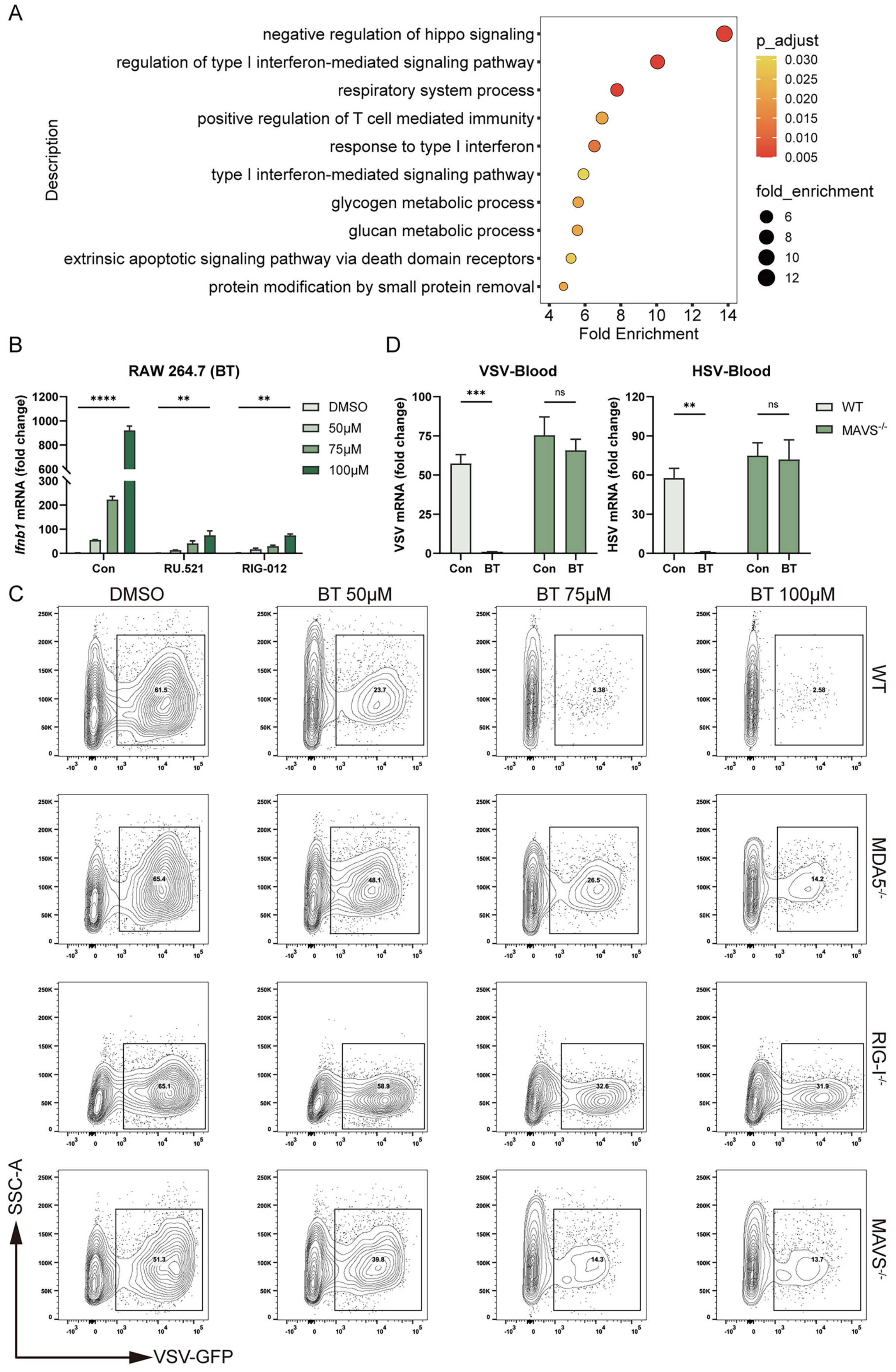
A
negative regulation of hippo signaling
regulation of type I interferon-mediated signaling pathway
respiratory system process
positive regulation of T cell mediated immunity
response to type I interferon
type I interferon-mediated signaling pathway
glycogen metabolic process
glucan metabolic process
extrinsic apoptotic signaling pathway via death domain receptors
protein modification by small protein removal
Description
Fold Enrichment
p_adjust
fold_enrichment
B
RAW 264.7 (BT)
Ifnb1 mRNA (fold change)
DMSO
50μM
75μM
100μM
Con
RU.521
RIG-012
D
VSV-Blood
HSV-Blood
VSV mRNA (fold change)
HSV mRNA (fold change)
WT
MAVS-/-
C
DMSO
BT 50μM
BT 75μM
BT 100μM
WT
MDA5-/-
RIG-I-/-
MAVS-/-
SSC-A
VSV-GFP

Figure EV5. Reactivated ERVs mediate the cytosolic dsRNA-sensing pathway.

(**A**) Gene Ontology terms enriched among BT-reactivated ERV loci. (**B**) RT-qPCR of *Ifnb1* in RAW 264.7 cells after BT treatment (100 μM, 12 h) with or without cGAS inhibition (RU.521) or RIG-I inhibition (RIG-012). (**C**) Representative density contour plot images of flow cytometry, the treatment was the same to Fig. 5H. (**D**) Viruses RNA levels in blood measured by RT-qPCR, the treatment is the same to Fig. 5I,J. Data are shown as mean ± SEM. N.S., not significant, $P > 0.05$; *$P < 0.05$; **$P < 0.01$; ***$P < 0.001$; ****$P < 0.0001$.

 © The Author(s)

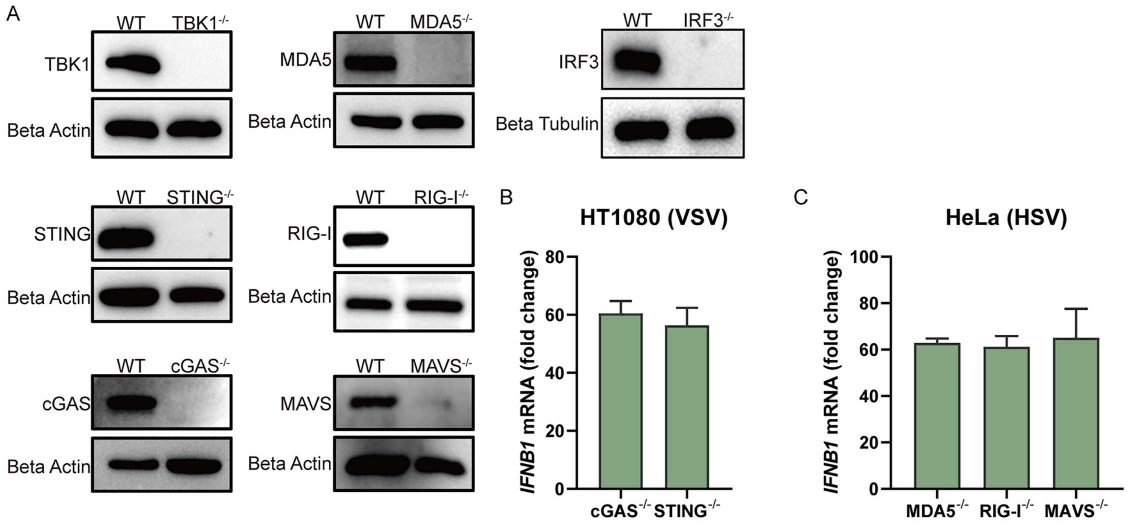

Figure EV6. Knockout cell lines.

(**A**) Western blot analyses of all knockout cell lines used in this study. (**B**, **C**) RT-qPCR to test the functions of knockout cell lines. Data are shown as mean ± SEM. N.S., not significant, $P > 0.05$; *$P < 0.05$; **$P < 0.01$; ***$P < 0.001$; ****$P < 0.0001$. Western blot quantifications are presented in Appendix Fig. S2.
